# Slaughter of Girl Babies

**Published:** 1892-01

**Authors:** 


### SLAUGHTER OF GIRL BABIES.

In China, tens of thousands of recently born girls among the poorer classes are thrown out to perish, and at Shanghai I saw a tower formerly used to facilitate this infanticide, says Dr. Joseph Simms, who has recently returned from an extended trip of the Flowery Empire. It is practiced in every part of China, but especially in the interior and in the Loess district. As soon as we get many miles from the coast it is usual to see near a joss house or place of worship, a small stone tower from ten to thirty feet high, with no door, but a hole in one side reaching into a pit in the centre.

The children that parents wish to get rid of are thrown into this hole, and quicklime soon consumes the little forms. It is said that the priests take charge of this cruel work. It has been estimated that every year 200,000 female babies are brutally slaughtered in the Empire. One Chinaman, being interrogated about the destruction of his recently born girl, said: "The wife cry and cry, but kill allee same."

In every large city in China there are asylums for the care of orphans, supported and conducted by foreigners, who save yearly from slaughter tens of thousands of female infants. At Han-Kow, which is 600 miles inland, I visited a Roman Catholic orphanage for children that have thus been cast out to perish Mother Paula Vismara, the Lady Superior of this institution, informed me that she had received seven that day, and one day thirty were brought in.

Of course these had never been consigned to a baby tower. Sometimes they are found wrapped in paper and left at the edge of the river. Sometimes they are buried alive by the father, but while yet living are dug up by some one else and brought to this institution. Several women are employed by the Mother Superior in looking about for the little victims. Upward of a thousand are received every year. Many of them, of course, die soon after from the exposure and neglect they have suffered through being abandoned, and many are boarded out by the institution in the town.

Those who accept the charges have to bring the children once a week for inspection, and then, all being right, they receive the pay for maintaining them. This is an Italian charity, and one of the most estimable in China. During the twenty-three years of its existence it has saved the lives of say twenty-five thousand to forty thousand children, of whom a fair proportion have grown to womanhood. It received considerable support from the European residents at Han-Kow, of whom there are about one hundred and twenty.

Those children who remain within the premises of the institution are fed and clothed, and when old enough, are taught to sew, make lace, knit stockings, and do other useful work. They never know where they come from or who their parents were. When they are four years of age their feet are bandaged, according to the general custom of all classes in China, to keep them small, as that increases their chances of marriage.

